# Medical Association of Georgia

**Published:** 1893-05

**Authors:** 


### GEORGIA STATE MEDICAL ASSOCIATION.

PROCEEDINGS OF THE FORTY-FOURTH ANNUAL MEETING, HELD AT AMERICUS APRIL 19TH, 20TH AND 21ST, 1893.

### FIRST DAY—MORNING SESSION.

The Association was called to order by the President, Dr. A. A. Smith, of Hawkinsville, at 10 a. m.

Prayer by the Rev. A. M. Williams.

Address of Welcome by Mayor A. S. Cutts, responded to on behalf of the Association by Dr. Mark H. O'Daniel, of Macon.

President's Annual Address, by Dr. A. A. Smith.

President Smith said that Georgia had her boards of education, her boards of trade, and her various other boards, but she had no board of health. The sanitary condition of her two million inhabitants, dwelling within the borders of 137 counties, should be looked after and cared for in an official way, and a board of health composed of the best medical talent in the State, should be established for that purpose. He knew of nothing that would be more conducive to the protection of the general health and property of the people than the permanent establishment of a State Board of Health. He also recommended the permanent establishment of a State Board of Examination, and said no one should be allowed to practice medicine within the borders of the State until he shall have passed a rigid and satisfactory examination under this board, and received therefrom a certificate of proficiency. This would drive away quacks, and thereby not only protect the health and the lives of the people, but also the honor and the dignity of the profession. Furthermore, the curriculum of medical institutions in Georgia should be raised to a higher standard. Noth ing less than the three course system should be tolerated, and each of these courses should be made broader, deeper, and more comprehensive. With a State Board of Health and a State Board of Examination, permanently established, and the curriculum of the Georgia medical schools raised to a higher standard, the profession would greatly accelerate the onward and upward progress of such a cause, and as it moved up onward and upward, higher and higher, mantled with the glory of its past and crowned with the splendor of its future God's truth would bloom upon its brow and God's approbation rest upon its work.

DR. C. C. HART, of Cross Keys, read a paper entitled, "Shot-Gun Prescriptions," in which he asked whether it was right to make what is known as "Shot-gun Prescriptions." Personally, he thought it was not, because it increased the difficulty of prescribing, and made the thereaputic action of medicines more uncertain and consequently less safe for the patient.

DR. J. I. DARBY, of Americus, contributed a paper entitled, "Puerperal Septicaemia and its Treatment."

FIRST DAY—AFTERNOON SESSION.

Paper by DR. J. M. HEAD, of Zebulon, entitled, "Puerperal Eclampsia with a Report of Cases."

True eclampsia might differ from epilepsy clinically, but the paroxysm of each were essentially the same. We find in eclampsia occasionally premonitory symptoms, which if properly interpreted by the obstetrician, will put him upon his guard and sometimes enable him to ward off the attack. However, in a considerable number of cases the symptoms are so slight as to escape detection, and suspicion is not aroused until severe convulsions are on. Hence we find it is of practical importance to bear well in mind the most common of the symptoms which are associated with cerebral troubles, such as severe headache either general or unilateral, spots before the eyes, dizziness, blindness, vertigo or impairment of the intellectual faculties which are occasionally observed. These symptoms are sufficient to excite the most serious apprehensions and lead us to make examinations of their causes.

So eclampsia should be considered of a fluctional nature through reflex influences, as chorea is an occasional feature of pregnancy. For the purpose of elucidation the speaker mentioned the prominent disorders of the pregnant stage.

DR. J. G. HOPKINS, Thomasville, read a paper on "Contagiousness of Consumption." The speaker said he had joined the growing army which placed tuberculosis in the category of contagious diseases, and his experience with this disease during nineteen years of investigation in Thomasville—which place is a resort for consumptives—bore him out in his opinion, and made him a willing subject of the great and erudite Koch. He does not doubt but that all men women and children, at some time or times receive into their air passages the tubercle bacilli, but fortunately the great majority possessed the power of repelling them and throwing them off, they did not find that soil, so to speak, which is adapted to their growth.

Dr. Hopkins emphasized the danger that lurks in sleeping cars, in carpets, bedding, clothing and in the walls of apartments occupied by consumptives, which have not been properly renovated and rendered harmless by antiseptic measures.

Consumptives should be forced to provide for the destruction of sputa. Whenever situated so as not to expectorate directly into a germicide or the fire, they should use some means of conveying the sputa to the germicide or the flames. The bacilli should never be allowed to dry up and impregnate the air, as is now done through ignorance of possible result. Numerous experiments by leading medical authorities have proven beyond doubt that consumption is an inoculable disease, and so rapidly is the throng of converts growing that the speaker would not be surprised if, even in his day, resorts now soliciting the patronage of the consumptive will be quarantining against him.

DR. J. MACFADDEN GASTON read a paper entitled, "Science in Medicine and Surgery." He said at this time the medical profession was undergoing a most interesting transition from the extreme views which had been held by some in regard to the employment of germicides in surgical practice. There was a time within the past decade when it was deemed to be scientific and progressive to use antiseptic measures of the most energetic kind in all operations, whether there was a septic element to combat or not. But thanks to the mature investigation of the effects of germicides upon normal structures by bacteriologists for this class of work, it has been demonstrated that the so-called antiseptic agents are capable of setting up septic processes in healthy tissues. The tables are now turned, and instead of a surgeon being compromised by eliminating germicides from his surgical procedures in ordinary cases, and confining his irrigation of recent wounds to simple sterilized water, it is he who departs from this course by the employment of solutions impregnated with toxic agents who is held responsible for the consequences of their absorption. In case there is a healthy and normal state of structures involved in a cutting operation, there can be no indication for antiseptics, and their use is only calculated to do harm by absorption, and the best men in the profession have ceased to employ them. The most energetic and hence the most likely to cause mischief is corrosive sublimate. Dr. Gaston quoted Dr. Vance, of Louisville, as saying that it was beyond all doubt that the present position of bichloride was rather against its use, and that it did more harm than good, as demonstrated by Welch and Abbott.

DR. MARK H. O'DANIEL, of Macon, read a short paper on "Multiple Neuritis Alcoholic," with report of case. The patient was thirty-six years of age, fleshy, short build, short neck, florid complexion, a bilious temperament, and a bookkeeper by occupation. The patient had taken but little exercise for quite a long while except in the elbow joints. He came of a nervous ancestry, an aunt having died of climacteric mania, and other relatives who had been insane for a short time, showing beyond doubt a predisposition on part of the patient to nervous trouble, which, if it had not existed, the use of alcohol might not have precipitated the disease in question. The patient drank from one to two pints of whiskey a day, that is during twenty-four hours. He had all the symptoms of such cases. Under appropriate treatment the patient made a good recovery.

Dr. Willis F. Westmoreland, of Atlanta, followed with a paper entitled, "Syphilis from a Sociological Standpoint," in which he said that year after year, particularly in the clinics, syphilitic subjects, a great many of them women, came before him suffering from this disease in all its stages, from the original lesion to the worst ravages of the second and tertiary stages, with mucous patches and syphilitic ulcers covering them. And when he found upon investigation, many of them servants, occupying various household positions as chambermaids, nurses, cooks, waitresses, etc., he conld not but think that God protects the family exposed to the baleful contamination of their service, and particularly the poor little defenseless ones whom they nurse, and with whom their relations are so intimate. Any one who has not investigated the subject will be overwhelmed with surprise at the number of cases acquired otherwise than by sexual intercourse, and the various means by which it has been transmitted. Physicians and dentists are themselves exposed, and likewise expose their patients to these dangers. Thousands of physicians and midwives in the practice of their calling, in examining and operating on syphilitic subjects, or by post-mortem, are exposed. Dentists by having their fingers scratched against a rough tooth or instrument, while working on a patient whose mouth is filled with mucous patches. On the other hand, many more patients are infected by the carelessness of physicians.

### SECOND DAY—MORNING SESSION.

Dr. W. H. Elliott, of Savannah, addressed the Association on "Elastic Constriction as a Haemostatic Measure." His remarks being a review of Senn's method. He said the Association was familiar with Esmarch's method, a "bloodless operation." Esmarch was not the inventor of the bloodless operation, but surgeons were indebted to his genius for improving its technique and for making this established method a popular procedure in surgery. The speaker then stated the two grave objections against the method as used by Esmarch, as advanced by Dr. Senn. The first objection was that forcible compression of the blood out of the tissues by an elastic bandage was liable to send into the surrounding tissues the elements of microbic and malignant diseases. Cancer cells may thus be scattered and disseminated through the system, or the cells of pus or of tuberculosis might be sent abroad to do damage elsewhere. The second objection raised was that the constricting of the limb with a tube or a narrow bandage at one point was liable to do injury first to the muscle and secondly to the nerve. Dr. Elliott had used Esmarch's method as modified by Dr. Senn with great satisfaction.

DR. O. H. BUFORD, of Cartersville, reported a rare case in obstetric practice, showing hour glass contractions on foetus.

DR. W. P. WILLIAMS, of Blackshear, read a paper entitled, "Persistent Remittent or So-called Typho-Malarial Fever." The author gave an analysis of a few cases of persistent remittent fever, touching its diagnosis and differential diagnosis from typhoid fever especially. He presented the clinical side of the matter, as his investigations extended no further. He presented three forms for consideration. First, the abortive form or such as are immediately amenable to treatment. Second, the continued form with low temperature. Third, the continued form with high temperature.

The value of a diagnosis between these maladies was apparent when we come to the subject of treatment. The treatment which the speaker had followed, which had proven reasonably successful in persistent remittent fever, was the antimalarial preparations, principally cinchona and its derivatives, and arsenic, with cholagogue cathartics (most frequently called calomel) at stated intervals, applying other medicines as they were indicated. This trreatment, while very beneficial in a large number of malarial cases, he considered utterly useless and even dangerous in typhoid cases.

DR. M. L. CURRIE, of Mount Vernon, read a paper entitled, "Periproctitis with an Abscess and Report of a Case." He said periproctitis was one of those infrequent inflammatory diseases which the general practitioner might at any time be called to treat, and which he might fail to recognize until much damage to his patient ensued. It is usually suppurative in character, but a cure may be effected by absorption, even after a distinct tumor is formed. Of the cause of the disease but little can be said. It may result from traumatism, foreign bodies, extension of adjacent inflammatory processes, or any structural disease involving the mucous membrane of the rectum. The manner of its extension and the course of the morbid processes excited, are identical with those seen in perityhplitis following typhlitis. The prognosis depends much upon the time when a diagnosis is made, the treatment of both the inflammation and the abscess as well as the physical condition of the patient. When the vitality is low and the abscess is high up in the pelvic cavity, or when the patient is tuberculous, it is unfavorable, when otherwise we may hope for recovery. The author then gave the diagnostic symptoms and the treatment in connection with the case he reported.

DR. R. R. KIME, of Atlanta, followed with a paper on "Ectopic Pregnancy, its Pathology, Symptoms and Treatment." After dwelling upon the pathology and symptoms, the author summarized the treatment as follows :

1. In primary or secondary intra-peritoneal rupture of ectopic sac operate by cœliotomy at once.

2. Before primary rupture with diagnosis of tubal pregnancy extirpate cyst, tube and ovary..

If diagnosis could be made in first three or four weeks of pregnancy then electricity might accomplish good results.

3. With extra-peritoneal primary rupture into broad lignment in early weeks of gestation producing pelvic hæmatocele, wait for absorption of effused blood; if it fails, then perform cœliotomy or vaginal drainage.

4. If child survives primary rupture of tube into broad ligament, do not commit murder, keep a clean conscience toward God and man, let foetus live, keeping patient under strict observance ready to operate at any time when life of patient demands it, or foetus has at least reached a viable period. By waiting for development of foetus and cyst as Tait claims, the peritoneum is pushed up on the side in which they are located, so that in later months of pregnancy a lateral incision will enter cyst, and foetus may be extracted without entering abdominal cavity.

5. Surgical interference should be the rule in all cases of ectopic pregnancy reaching full term even after spurious labor and death of foetus.

DR. LUTHER B. GRANDY, of Atlanta, read a paper entitled, "A Board of Medical Examiners; the State's Medical Duty," in which he suggested in November last the advisability of taking steps toward the establishment of a board of medical examiners in Georgia. The question under discussion was one which touched every home in Georgia, into which sickness or accident would surely come at one time or another. There were two classes of so-called doctors from whom the people of this and every State need to be protected. One was the ignorant practitioner without capacity, the other was the unprincipled charlatan without conscience. The absence of such a board and the fact that a diploma was a lisence was but throwing down the bars to all the incompetent and fraudulent who were being rejected in other places. If the medical profession in Georgia was to be made up largely of those who had been refused by other States on account of incompetency or dishonesty, then it was time we were entering our protest at once. There had been nearly 400 rejections by the Virginia and North Carolina boards within the last seven years. What became of all those persons who were examined and declined by the boards of New Jersey, Virginia, North Carolina, Florida, Alabama, Illinois, Minnesota and other States? Our neighbors, Alabama, South Carolina and Florida, have rejected respectively, 20, 29 and 30 per cent. of their applicants. Where do these people go? As for the quacks, when driven from one place they quickly take refuge in another. They change their sky but not their affections. (*Coelum non animum, mutant.*) Dr. Grandy closed by saying that if Georgia would like to divorce the right to practice medicine from the empty honor of having a diploma, then let there be established a Board of Medical Examiners who shall be untrammeled by any college connections, and who shall determine whether a given applicant is qualified to practice medicine in Georgia.

DR. ARTHUR C. BLAIN, of Macon, contributed a paper entitled, "The Practice of Medicine in Georgia." He called attention to the want of laws governing the practice of medicine in the State. He also emphasized the importance of creating a State Board of Medical Examiners. Georgia was made a dumping-ground for all who failed to pass the requisite examination to obtain a license to practice in Virginia, Florida or Alabama. State Boards not only afforded protection to the people from charlatans and unqualified practitioners, but exerted a salutary influence towards elevating the educational standard of the medical profession. The speaker recommended that a committee be sent to Atlanta during the next session of the legislature to assist in formulating and passing some good law that would meet the requirements of the case.

DR. J. C. AVARY, of Atlanta, followed with a paper on "State and Municipal Hygiene."

The three papers were ably discussed by Drs. C. D. Hurt, R. O Engram, Willis F. Westmoreland, W. H. Elliott, F. W. McRae, M. B. Hutchins, and a committee of one from each congressional district appointed for the purpose previously outlined.

SECOND DAY—AFTERNOON SESSION,

DR. C. D. HURT, of Atlanta, read a paper entitled, "Etiology of Puerperal Eclampsia, its Treatment, with Report of some Typical Cases." After a careful study of various authors and summing up his observations and experience at the bedside, the author felt warranted in offering the following conclusions: 1. That eclampsia gravidarum in every case is the result of some influence which is directly or indirectly exerted upon the nervous system causing a derangement of its functions. 2. That such influences may exist singly or be co-operative, and that such influence may be direct violence or mechanical pressure. 3; That one or more of these influences may reach the nervous system by absorption or through the circulation. 4. That by chemical changes wrought under certain conditions in the system, toxic elements may be created and proved deleterious. That in every case irritation may and does exert an influence which materially aids in bringing on a seizure, and that in many cases this irritation intensified by protracted labor, hard pains, unyielding os and exhaustion of the nervous system develops eclampsia.

DR. H. MCHATTON, of Macon, read a paper entitled, "Four Women who Refused Oophorectomy and their Subsequent Histories." The author gave a brief *resume* of the histories of the only four women that he had ever known who refused the operation. The time that had elapsed since it was advised varied from eighteen months to twelve years. The speaker made the point that in each case the operation was advised and urged by gynecologists of standing both North and South, consequently they must have been convinced that they were cases demanding operative interference. In a practice of twelve years he had occasion to recommend the removal of the appendages once, excepting cases of ovarian tumors, and that one proved to be a case of pyosalpinx. Several of his patients had drifted into other hands and had oophorectomy performed, and as far as he could learn had been disappointed in the results each time. Thc best men in this special line of work were doing the operation less and less each year. Their place was being amply filled by lesser lights, with smaller numbers of individual cases, but with a yearly aggregate that is terrible to contemplate. By what combination of circumstances could one man, to fame unknown. in a small interior city, and in a short space of time, find 144 cases demanding abdominal section.

DR. C. H. PEETE, of Macon, contributed a paper on " Partial Tenotomy, a Radical Cure for Heterophoralgia." The author finds that the majority of cases suffering from heterophoralgia are those of young adult life, or that they have been sufferers since that time. He accounts for it by the fact that we know in order to cause it the patient must concentrate his vision on an object, and the length of time of this concentration governs the intensity of suffering. In children the vision is never fixed on any point for a length of time, consequently the symptoms are not brought on. In young adults who have acquired the habit of hard study and constant close work, it occurs frequently, and we will find in most cases that it commenced with the time of their beginning to work or in a short time after. The muscle tests should be applied to every case that came to the oculist for refraction work, and when he finds these deviations existing he should resort to the radical relief, partial tenotomy.

DR. F. W. MCRAE, of Atlanta, read a paper entitled, "Stone in the Bladder," with report of cases. He dealt with the etiology, symptomatology and treatment of stone in the bladder, purposely avoiding extensive details and references to authorities. In one of the cases which the author reported the symptoms were characteristic of stone in the bladder, although no stone was found. The operation was a brilliant success in relieving the patient of a most violent cystitis of long standing. The author then dealt with the methods of treating stone in the bladder, and said that no method had so wide a range of applicability and as low a death rate as litholapaxy (Bigelow's operation.) He had had, however, no personal experience with it, as the cases thus far presenting themselves to him were such as might be best treated by other procedures. This operation offered much the best results except in very young children, very large or very hard stones, or where there was co-existing tight organic strictures or enlarged prostate of such character as to prevent the introduction of the lithotrite, or where there was a violent cystitis associated with stone. For large stones associated with prostate or vesical tumors the high operation was undoubtedly far superior to the perineal operation. Small stones associated with stricture of the deep urethra or violent cystitis were best treated by the median operation.

DR. W. R. GOOGE, of Abbeville, reported a case of fracture of the skull, with protrusion of the brain substance and removal of same.

THIRD DAY—MORNING SESSION.

DR. J. B. S. HOLMES, of Rome, read a lengthy paper on "The Technique and After-Treatment of Ovariotomy." The author offered the paper as an *expose* of the methods adopted and successfully used by him in his abdominal work. He submitted the paper with the earnest hope that it might elicit a full and free discussion from all those interested in this line of work. By such discussions our methods may be compared and those adopted that promise best results to our patients. No one could truthfully say that in every particular his method was the best. While we might in the main agree, yet upon slight and seemingly indifferent details might hinge the successful issue of our cases. He stood ready and willing to take up any suggestions from his brother surgeons if he could get better results by so doing. The author then took up each step *seriatim* of the technique and after-treatment.

DR. FRANK M. RIDLEY, of LaGrange, then delivered the Orator's Address. He selected for his subject "Woman's Relations to the Practice of Medicine."

DR. M. B. HUTCHINS, of Atlanta, contributed a paper entitled, "Mechanical Treatment of some Skin Anomalies." The author dwelt upon some of the various mechanical means of removing anomalies of the skin. The destruction of superfluous hairs, of moles, birth-marks, and the various simple growths of the skin were described. In 1879, Dr. Mitchel, of St. Louis, successfully employed electrolysis for the destruction of the hairs in trichiasis. Dr. Hardaway, a dermatologist of the same city, followed the idea in the treatment of hypertrichosis. Various dermatologists had since reported upon its successful use. Hair upon the lip and face of women afforded the usual field of operation. Three to eight cells of a galvanic battery were used. To the negative pole a pencil-shaped needle holder containing a number 8 or 12 ordinary sewing needle is attached, while the positive pole is provided with the usual sponge electrode. The needle is attached to the negative pole for the reason that electrolysis through the positive will leave a black dot at the point of insertion of the needle. The needle is carefully inserted into the hair follicle, parallel to the root, until slight resistance is felt. The circuit is then closed and the current allowed to pass until a frothy substance, the size of a small pin head, fills the follicle, and a small area of blanching appears around the needle. If the needle has followed the course of the hair root and remained in the follicle, the pain is less than when the root sheaths are pierced, and the frothy substance is more abundant. If the hair is easily pulled out it is reasonably certain that there will be no regrowth, but if it pulls out with difficulty or breaks off, the current must immediately be reapplied. A pair of forceps made for the purpose are used to extract the hairs.

Dr. Hutchins then reported several interesting cases which he had treated successfully by electrolysis.

The following officers were elected: President, Dr. W. H. Elliott, Savannah; First Vice-President, Dr. G. T. Miller, Americus; Second Vice-President, Dr. H. McHatton, Macon; Secretary, Dr. Dan. H. Howell, Atlanta; Treasurer, Dr. E. C. Goodrich, Augusta.

The next annual meeting of the Association will be held in Atlanta.

